# Application of disitamab vedotin in the multiline treatment of EGFR mutation-positive lung adenocarcinoma with Her-2 overexpression

**DOI:** 10.3389/fonc.2024.1472545

**Published:** 2024-12-12

**Authors:** Meiling Lan, Tianyun Wang, Diexiao Luo, Yan Chen, Wei Liang, Rui Kong, Qichao Xie

**Affiliations:** ^1^ Department of Oncology, The Third Affiliated Hospital of Chongqing Medical University, Chongqing, China; ^2^ Department of Pathology, Daping Hospital, Army Medical University, Chongqing, China

**Keywords:** HER-2 overexpression, epidermal growth factor receptor, lung adenocarcinoma, disitamab vedotin, efficacy, multiline treatment, conservative treatment

## Abstract

**Objective:**

To explore the efficacy of c in the multiline treatment of late-stage lung adenocarcinoma with Her-2 overexpression and epidermal growth factor receptor (EGFR) mutations.

**Methods:**

We summarize the diagnosis and treatment of a female patient with EGFR 21L858R mutation combined with Her-2 overexpression in advanced lung adenocarcinoma, and analyze the effect of c in her treatment process.

**Results:**

The patient was diagnosed with lung adenocarcinoma 8 years ago. After first-line treatment, the lung lesions enlarged. Following second-line treatment 5 years ago, intracranial metastasis occurred. After third-line treatment 3 years ago, intracranial and lung lesions enlarged. New lesions in the lungs, liver, and spleen appeared after fourth-line treatment 32 months ago. Lung progression occurred after fifth-line treatment 29 months ago. Liver and lung progression occurred after sixth-line treatment 22 months ago. Lung progression occurred after seventh-line treatment 19 months ago. The patient underwent eighth-line treatment with disitamab vedotin (RC48) + lung radiotherapy + liver intervention 13 months ago. Currently, the patient’s condition is stable, with a good quality of life, and the efficacy assessment is stable disease (SD). Conclusion: Her-2 overexpression can occur in late-stage EGFR-mutant lung adenocarcinoma after multiline treatment. RC48 can achieve sustained remission in these patients.

## 1 Introduction

Lung cancer is the most common malignant tumour, with non-small cell lung cancer (NSCLC) accounting for 80% to 85% of all lung cancers (1). Targeted therapies against specific molecular mutations, such as epidermal growth factor receptor (EGFR), anaplastic lymphoma kinase (ALK), and other driver genes, have rapidly advanced, improving the prognosis and quality of life of NSCLC patients (2). Compared to chemotherapy, targeted therapy has prolonged progression-free survival (PFS) and overall survival (OS) times. However, due to tumour heterogeneity and genomic instability, the widespread occurrence of secondary resistance, such as secondary gene mutations and activation of alternative signalling pathways, has greatly reduced the cure rate of non-small cell lung cancer, leading to the emergence of corresponding drug resistance issues (3). The emergence of immunotherapy has changed the treatment landscape for advanced NSCLC; however, research has mainly focused on NSCLC patients who are negative for driver genes (4, 5). For patients who are resistant to EGFR-tyrosine kinase inhibitors (TKIs) and have no standard targeted therapy, clinical studies have further explored the efficacy of immunotherapy in patients after EGFR-TKIs resistance, providing new treatment options for patients resistant to EGFR-TKIs (6, 7). Currently, immunotherapy combined with chemotherapy has shown initial effectiveness (8, 9), and the combination of immunotherapy with doublet chemotherapy and anti-angiogenesis quadruple therapy has shown remarkable efficacy (6). The frequency of Her-2 gene abnormalities in NSCLC is lower than that of EGFR, but its tumour driving mechanism is clear, and it is sensitive to some targeted drugs, making it a current research hotspot. This article summarizes the diagnosis and treatment of a patient with late-stage lung adenocarcinoma with Her-2 overexpression and EGFR mutation, aiming to explore the treatment efficacy of the antibody-drug conjugate (ADC) disitamab vedotin. The report is as follows.

## 2 Materials and methods

### 2.1 General information

The patient, a 52-year-old female, was admitted to the hospital on September 8, 2021, due to “shortness of breath and increased fatigue after activity for 1 week.” The patient was diagnosed with lung adenocarcinoma at an outside hospital 8 years ago due to “persistent cough and shortness of breath for over 2 months.” After 38 months of first-line treatment, lung lesions enlarged. Twenty months into second-line treatment 5 years ago, intracranial metastasis occurred. Five months into third-line treatment 3 years ago, intracranial and lung lesions enlarged. Four months into fourth-line treatment 2.5 years ago, new lesions appeared in the lungs, liver, and spleen. Seven months into fifth-line treatment 2.2 years ago, lung progression occurred. Three months into sixth-line treatment, liver and lung progression occurred. Five months into seventh-line treatment, lung progression occurred. Thirteen months ago, the patient underwent eighth-line treatment with RC48, combined with lung radiotherapy and liver local interventional therapy. Currently, the patient’s condition is stable, with a good quality of life. This study was approved by the hospital’s ethics committee (Approval No: Coren Trial No. (4) of 2024), adhering to the principles of the Helsinki Declaration, and informed consent was obtained from the patient.

### 2.2 methods

#### 2.2.1 Genetic testing

Pathological paraffin-embedded (FFPE) tissue and blood samples from the patient were used for genetic testing. DNA was extracted using the Magnetic Bead-based FFPE DNA Extraction Kit (Guangzhou Meiji Biotechnology Co., Ltd., Guangzhou, China) and the Magnetic Bead-based Blood Genomic DNA Extraction Kit-T5C (Tiangen Biochemical Technology (Beijing) Co., Ltd., Beijing, China). Next-generation sequencing (NGS) was performed using a gene capture panel to detect mutations in 122 genes related to solid tumors (Wuxi Zhenhe Biotechnology Co., Ltd.).

#### 2.2.2 Immunohistochemistry

Specimens were fixed in 10% neutral buffered formalin for 24 hours, routinely dehydrated, embedded in paraffin, and sectioned at 3 μm. Hematoxylin and eosin (HE) staining was performed for microscopic observation. Immunohistochemistry staining was performed using an automated immunohistochemistry staining machine (Roche, Switzerland) and the UltraView Universal DAB Detection Kit (Ventana), purchased from Roche Diagnostics Products (Shanghai) Co., Ltd.

### 2.3 Statistical analysis

Count data were expressed as frequencies or percentages.

## 3 Results

### 3.1 Patient’s multiline treatment course

The patient’s multiline treatment course is shown in **Table 1**.

**Table 1:** Patient’s Multiline Treatment Course.

Stage	Time	Treatment Regimen	Treatment Efficacy	PFS(months)
1st Line	2016.2-2019.4	Gefitinib 250mg po qd	Lung PD	38
2nd Line	2019.4-2020.12	Osimertinib 80mg po qd	Brain PD	20
3rd Line	2020.12-2021.5	Osimertinib 80mg po qd + (Pemetrexed Disodium 745 mg iv d1 + Cisplatin 30 mg iv d1-3) x 4 cycles	Brain, lung PD	5
4th Line	2021.6-2021.9	Osimertinib 80mg po qd + (Bevacizumab 400mg iv d1 + Pemetrexed Disodium 750mg iv d1) x 3 cycles	Lung, liver, spleen PD	4
5th Line	2021-9.2022.4	Afatinib 30mg po qd + (Paclitaxel Liposome 180mg iv d1 + Nedaplatin 50mg iv d1-2) x 3 cycles. Liver local radiotherapy PGTV 69Gy/30F, Whole-brain radiotherapy PGTV 50Gy/20F, PTV 40Gy/20F	Lung PD	7
6th Line	2022.4-2022.7	Pyrotinib 400mg po qd	Lung, liver PD	3
7th Line	2022.7-2022.12	Anlotinib 12mg po d1-14 + Toripalimab 240 mg iv q3w	Lung PD	5
8th Line	2022.12-present	Disitamab vedotin 120mg iv q3w x 13 cycles. Lung radiotherapy PGTV 69Gy/30F, Liver local interventional therapy 2 times	PR	13

(PD, Progressive Disease; PR, Partial Response; PGTV, Planning Gross Target Volume; PTV, Planning Target Volume; Gy, Gray).

### 3.2 Genetic testing results

On 18 January 2016, genetic testing (tissue) showed an EGFR 21 L858R mutation. On 22 April 2019, genetic testing (peripheral blood) was negative for T790M mutation, with no relevant driver gene mutations detected. On 2 April 2020, genetic testing (peripheral blood) confirmed the EGFR 21 L858R mutation with a mutation abundance of 0.29%. On 14 September 2021, genetic testing (tissue and blood) showed an EGFR p.L858R mutation with a mutation frequency of 56.43%, and ERBB2 gene amplification with a copy number change of 26.07.

### 3.3 Pathology and Her-2 testing results

On 18 January 2016, a percutaneous lung biopsy (left upper lung) revealed adenocarcinoma. Immunohistochemistry did not detect Her-2. On 14 September 2021, a percutaneous lung biopsy (left lung) again showed adenocarcinoma. Immunohistochemistry did not detect Her-2. On 7 December 2022, a percutaneous lung biopsy (left lung tissue) confirmed adenocarcinoma. Immunohistochemistry showed Her-2 (3+).

### 3.4 Imaging results

**Figure 1:**
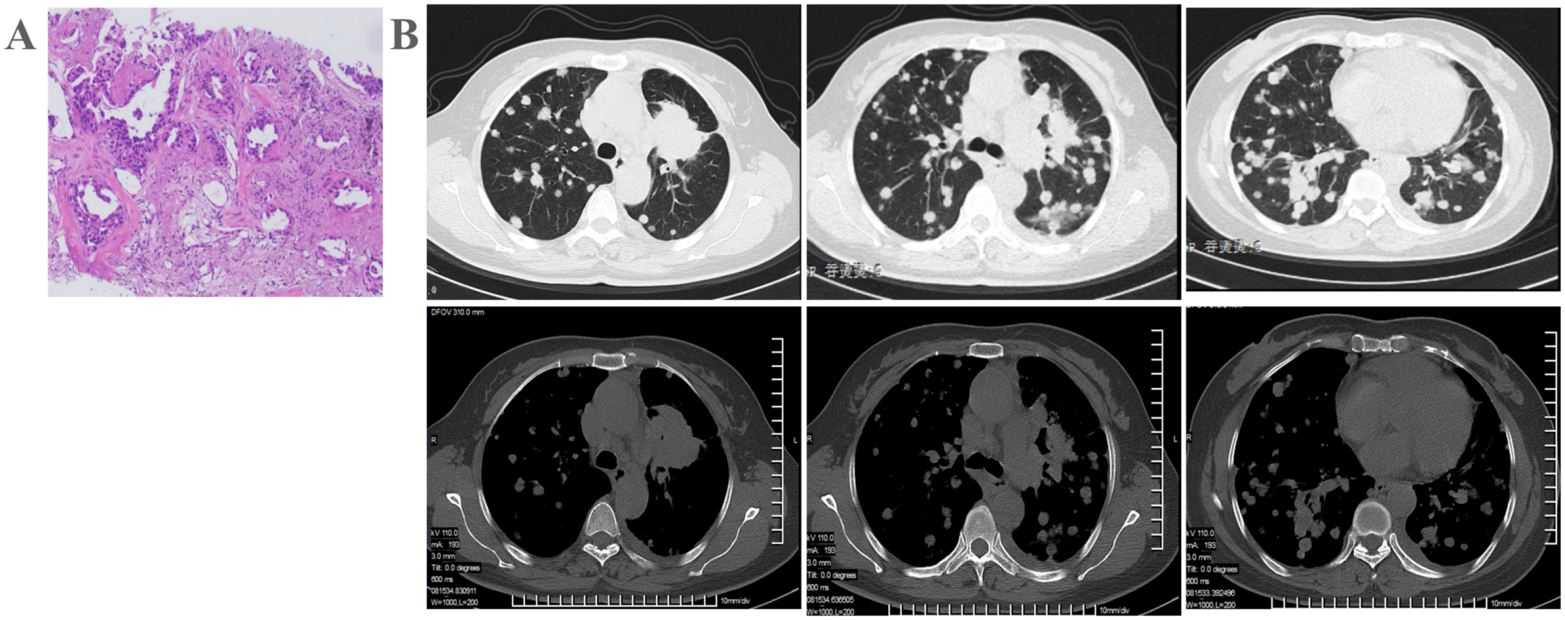
Patient examination before treatment. **(A)** Chest CT indicates left upper lung cancer with a high likelihood of bilateral lung metastasis, and multiple enlarged mediastinal lymph nodes. **(B)** Pathological biopsy confirms lung adenocarcinoma.

**Figure 2:**
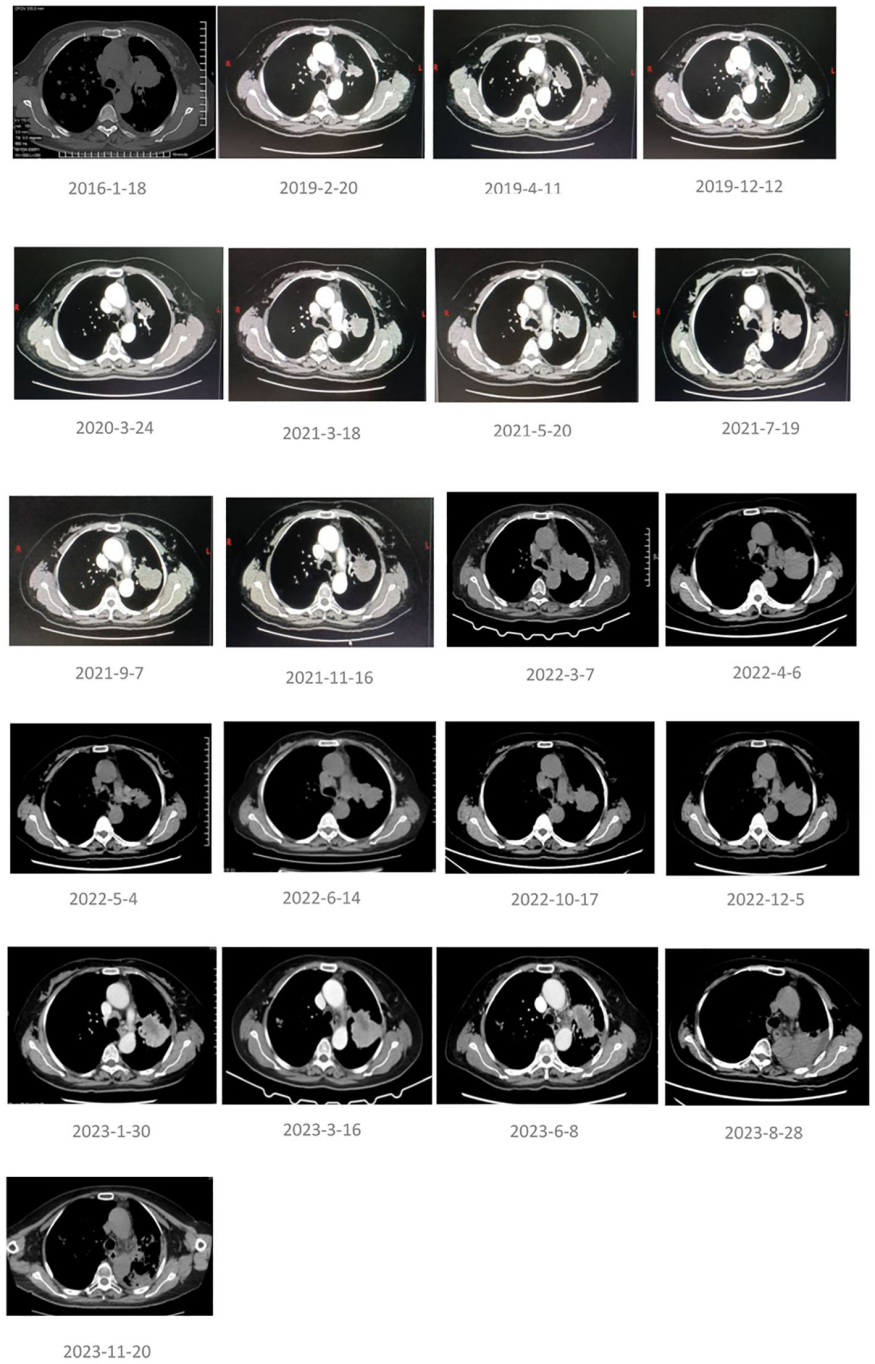
Chest CT images at key points during the patient’s treatment process.

**Figure 3:**
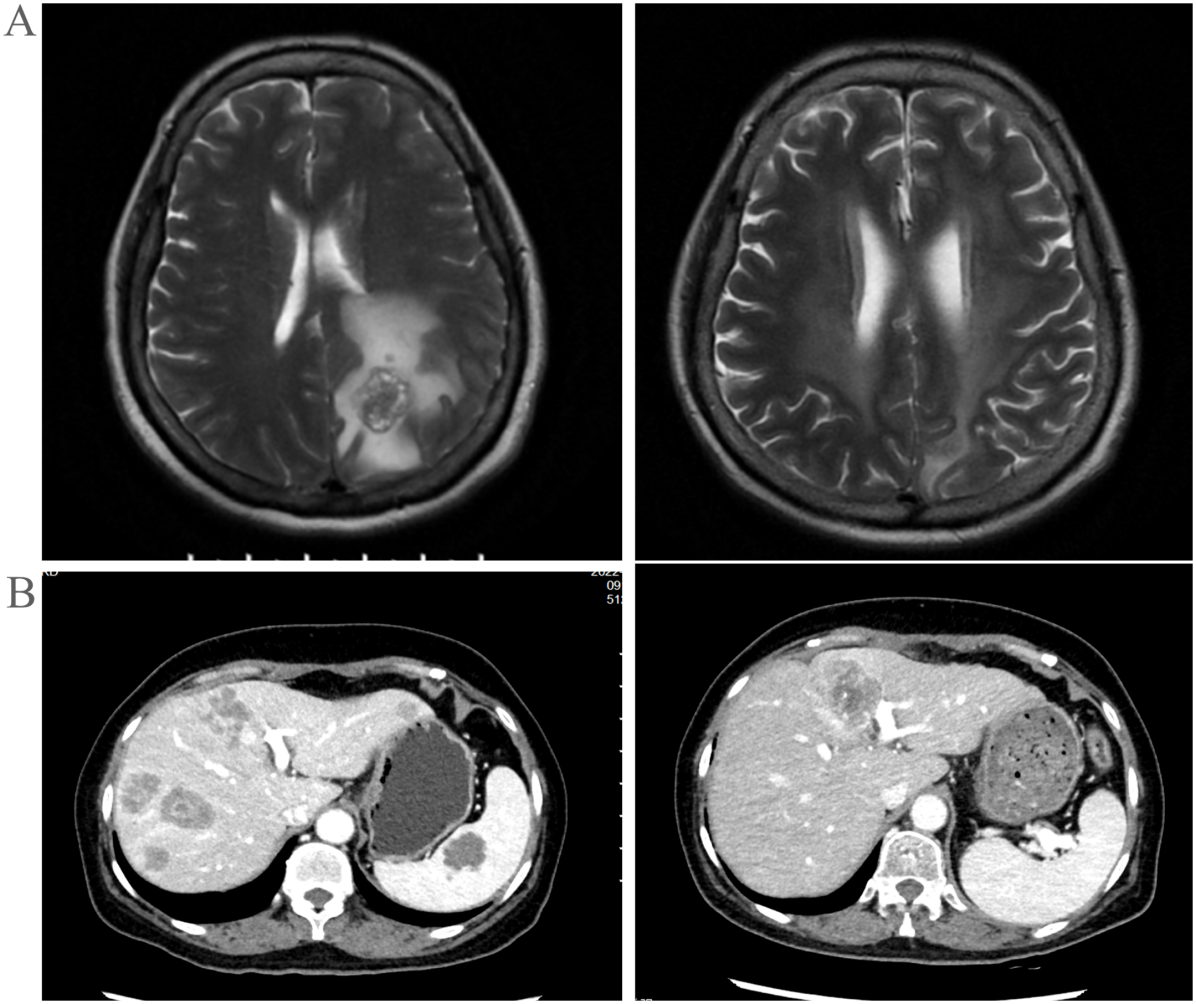
**(A)** Extra-pulmonary organs: head, MRI images before and after treatment. **(B)** Extra-pulmonary organs: liver, CT images at key points during the treatment process.

The pathology and imaging results at the initial diagnosis on 18 January 2016 are shown in **Figure 1**. The lung imaging results during the treatment period are shown in **Figure 2**. The imaging results of extracorporeal organs (brain, liver, spleen) before and most recently during RC48 treatment are shown in **Figure 3**.

### 3.5 Efficacy

Since the start of eighth-line treatment, the patient has achieved 13 months of sustained remission. As of 20 January 2024, the patient’s overall survival (OS) time has reached 8 years.

## 4 Discussion

Most patients with non-small cell lung cancer (NSCLC) are diagnosed at an advanced stage and have a poor prognosis (10). In recent years, with the discovery of driver genes in NSCLC, especially adenocarcinoma, and advances in drug development, the survival of patients with advanced NSCLC has significantly improved, marking the advent of the targeted therapy era and providing new treatment options for NSCLC (11). Drugs such as gefitinib, dacomitinib, and osimertinib, EGFR-TKIs, have been approved by the FDA for the treatment of NSCLC with positive driver genes. However, the clinical efficacy of EGFR-TKIs is greatly limited by inevitable resistance, with resistance mechanisms including Her-2/Her-3/c-Met amplification and receptor tyrosine kinase-related bypass mechanisms, with Her-2 being the most representative (12).

Her-2 is a tyrosine kinase receptor in the ERBB/Her family and, together with other family members like EGFR, activates downstream signal transduction. Abnormalities in the Her-2 gene are closely related to the severity of many epithelial cell cancers, with tumours exhibiting strong metastatic and invasive capabilities, poor sensitivity to chemotherapy, and a high tendency for recurrence. Her-2 mutations and amplifications are associated with female gender, Asian ethnicity, non-smoking status, and poorly differentiated adenocarcinoma histology. In NSCLC, patients with Her-2 positivity have a shorter survival period compared to the general population (13). The forms of Her-2 variations in NSCLC primarily include mutations (2%–4%), amplifications (10%–20%), and overexpression (6%–35%). NSCLC resulting from Her-2 mutations, amplifications, or overexpression is referred to as Her-2 positive NSCLC (14).

In this study, the patient underwent a fourth genetic test (tissue and blood), which revealed an EGFR p.L858R mutation with a mutation frequency of 56.43% and ERBB2 gene amplification with a copy number change of 26.07. Afatinib, a pan-Her (EGFR/Her-1, Her-2, and Her-4) inhibitor, selectively and irreversibly binds to its HER family receptor targets, providing long-lasting inhibition. According to the LUX-Lung5 study (15), the progression-free survival (PFS) and objective response rate (ORR) in the afatinib plus paclitaxel group were significantly higher than those in the monotherapy chemotherapy group, with PFS (5.6 months vs. 2.8 months, HR=0.60, P=0.003) and ORR (32.1% vs. 13.2%, P=0.005) showing marked improvement. After multidisciplinary discussion and considering the patient’s financial situation, the fifth-line treatment was chosen as afatinib combined with paclitaxel liposome and nedaplatin for three cycles, followed by liver and brain radiotherapy for further tumour control. After seven months, the efficacy evaluation indicated disease progression.

Her-2 antibodies such as trastuzumab have not significantly improved efficacy compared to traditional chemotherapy, and pan-HER inhibitors like dacomitinib and afatinib have shown unsatisfactory results. However, some new TKIs have shown initial advantages. A phase II prospective clinical study (ChiCTR180000262) indicated that pyrotinib treatment for Her-2 amplified populations had an ORR of 22.2%, a median PFS of 6.3 months, and a median OS of 12.5 months. Additionally, 30.8% of cases with progression on EGFR-TKIs responded to pyrotinib, and the ORR for patients with brain metastases was 40% (16). After one month of pyrotinib treatment, the tumour size was significantly reduced, with the efficacy evaluated as partial response, but after three months, liver and lung progression recurred.

According to the 2022 Chinese Expert Consensus on Immunotherapy for Advanced NSCLC with Driver Genes, for patients with extensive progression after resistance to EGFR-TKIs and in the absence of effective targeted treatments, the use of immune checkpoint inhibitors (ICIs) is recommended. Among the recommended regimens, ICIs combined with chemotherapy and anti-angiogenesis treatment, and ICIs combined with platinum-based chemotherapy, have substantial clinical evidence.For patients similar to those in this study, who have undergone multiple lines of treatment and cannot tolerate high-intensity therapy, ICIs combined with anti-angiogenic treatment is recommended (16). A real-world study in China (17) demonstrated that in second-line and subsequent treatments for recurrent NSCLC patients, the combination of toripalimab and anlotinib showed synergistic effects, significantly prolonging PFS compared to immunotherapy alone or single-agent chemotherapy. Therefore, the seventh-line treatment employed toripalimab combined with anlotinib. After five months, the efficacy assessment indicated disease progression. A repeat lung biopsy and immunohistochemistry revealed Her-2 (3+) status.

Antibody-drug conjugates (ADCs) have emerged as one of the fastest-growing areas in lung cancer treatment in recent years. Combining tumour cell-specific monoclonal antibodies (mAbs) with cytotoxic drugs, ADCs achieve both tumour cell targeting and cell-killing capabilities, positioning them as a promising future direction in cancer therapy. For Her-2 mutant NSCLC, ADCs have shown outstanding performance. In a phase II basket trial of T-DM1 (18), the ORR was 44%, with a PFS of 5.0 months, although the sample size was relatively small. T-DM1 demonstrated some clinical efficacy in NSCLC patients with Her-2 mutations and amplifications, but its effectiveness in Her-2 overexpressing NSCLC did not meet expectations. Trastuzumab deruxtecan (T-DXd) has shown remarkable results in treating Her-2 mutant NSCLC. Initial data from a phase I study reported an ORR of 72.7% and a PFS of 11.3 months (19). The phase II study (DESTINY-Lung01) showed an ORR of 55% and a PFS of 8.2 months (20). In the DESTINY-Lung01 study, for the Her-2 overexpressing (3+ or 2+) cohort, results presented at the 2022 ESMO conference indicated that the ORR assessed by ICR was 26.5% (cohort 1, 6.4 mg/kg) and 34.1% (cohort 1a, 5.4 mg/kg); the median PFS was 5.7 and 6.7 months, respectively, and the median OS was 12.4 and 11.2 months, respectively (21). While T-DXd shows significant promise in Her-2 mutant NSCLC, its efficacy in Her-2 overexpressing NSCLC is limited.

RC48 is a novel humanised anti-Her-2 ADC. It uses a Her-2 antibody as a targeting carrier, covalently conjugated to a small molecule toxin (MMAE) via a cleavable linker. In a phase II study of third-line treatment for locally advanced or metastatic Her-2 overexpressing gastric cancer or gastroesophageal junction cancer (22), the results showed an ORR of 24.4%, a median PFS of 4.1 months, and a median OS of 7.6 months. In patients with previously failed chemotherapy for Her-2 overexpressing locally advanced or metastatic urothelial carcinoma, the ORR with RC48 treatment was 50.0% (23). RC48 has demonstrated clinical benefits in the treatment of gastric cancer and urothelial carcinoma.A real-world retrospective study (24) included 23 patients with advanced solid tumours such as breast cancer, gastric cancer, colorectal cancer, and bladder cancer, with at least Her-2 immunohistochemistry 1+ expression and failure after at least one systemic chemotherapy. All patients received RC48 treatment (as monotherapy, combined with immunotherapy, or combined with radiotherapy). The ORR was 43.5%, and the median PFS was 6.0 months. Further stratified analysis showed that the ORR for the HER-2 low/medium expression (1+ or 2+) group was 37.5%, with a median PFS of 5.75 months. For the HER-2 high expression (3+) group, the ORR was 57.1%, with a median PFS of 7 months. In the RC48 combined with PD-1 inhibitor group, the ORR was 53.8%, with a median PFS of 8 months. In the group combined with local radiotherapy, the ORR was 40.0%, with a median PFS of 6.0 months. Current phase II/III clinical studies of RC48-ADC are ongoing for indications such as breast cancer, lung cancer, and cholangiocarcinoma. Considering the availability of the drug, the patient opted for RC48 treatment, during which the lesion assessment indicated partial response.

Considering that radiotherapy can reduce the tumour burden of local lesions and release tumour antigens, RC48-ADC targets Her-2 antigens on the surface of tumour cells, precisely identifying and destroying tumour cells. This can also lead to extensive antigen release from other metastatic lesions, thereby activating T-cell immunity and forming a “point-to-surface” treatment strategy. Therefore, RC48-ADC and radiotherapy may have a synergistic effect, achieving better therapeutic outcomes (25). Consequently, to better reduce the tumour, the patient underwent palliative radiotherapy of the left lung and received two sessions of liver interventional therapy. During treatment, the patient experienced mild adverse events, including grade II leukopenia and grade I anaemia, which improved after symptomatic treatment. No other treatment-related adverse events such as skin toxicity, neurotoxicity, cardiotoxicity, pulmonary toxicity, gastrointestinal toxicity and hepatic toxicity occurred during treatment. Currently, the patient’s PFS is 13 months, with an OS of 8 years.

In summary, the patient achieved long-term survival through multiple lines of treatment, including targeted therapy, chemotherapy, radiotherapy, immunotherapy, and antibody-drug conjugates, indicating that lung cancer treatment is progressing towards a chronic disease management approach. This study highlights that Her-2 positivity in NSCLC presents a challenging therapeutic target, with current clinical needs not yet fully met. ADCs show great potential in the standard treatment pathway for Her-2 mutant NSCLC patients. However, further exploration is needed regarding the biological nature, treatment strategies, and diagnostic standards of Her-2 amplification/overexpression in lung cancer.

## Data Availability

The original contributions presented in the study are included in the article/supplementary material. Further inquiries can be directed to the corresponding authors.
